# A Flow Cytometry-Based Screen of Nuclear Envelope Transmembrane Proteins Identifies NET4/Tmem53 as Involved in Stress-Dependent Cell Cycle Withdrawal

**DOI:** 10.1371/journal.pone.0018762

**Published:** 2011-04-14

**Authors:** Nadia Korfali, Vlastimil Srsen, Martin Waterfall, Dzmitry G. Batrakou, Vanja Pekovic, Christopher J. Hutchison, Eric C. Schirmer

**Affiliations:** 1 The Wellcome Trust Centre for Cell Biology and Institute of Cell Biology, University of Edinburgh, Edinburgh, United Kingdom; 2 Institute of Immunology and Infection Research, University of Edinburgh, Edinburgh, United Kingdom; 3 School of Biological and Biomedical Sciences, Durham University, Durham, United Kingdom; Brunel University, United Kingdom

## Abstract

Disruption of cell cycle regulation is one mechanism proposed for how nuclear envelope protein mutation can cause disease. Thus far only a few nuclear envelope proteins have been tested/found to affect cell cycle progression: to identify others, 39 novel nuclear envelope transmembrane proteins were screened for their ability to alter flow cytometry cell cycle/DNA content profiles when exogenously expressed. Eight had notable effects with seven increasing and one decreasing the 4N∶2N ratio. We subsequently focused on NET4/Tmem53 that lost its effects in p53^−/−^ cells and retinoblastoma protein-deficient cells. NET4/TMEM53 knockdown by siRNA altered flow cytometry cell cycle/DNA content profiles in a similar way as overexpression. NET4/TMEM53 knockdown did not affect total retinoblastoma protein levels, unlike nuclear envelope-associated proteins Lamin A and LAP2α. However, a decrease in phosphorylated retinoblastoma protein was observed along with a doubling of p53 levels and a 7-fold increase in p21. Consequently cells withdrew from the cell cycle, which was confirmed in MRC5 cells by a drop in the percentage of cells expressing Ki-67 antigen and an increase in the number of cells stained for ß-galactosidase. The ß-galactosidase upregulation suggests that cells become prematurely senescent. Finally, the changes in retinoblastoma protein, p53, and p21 resulting from loss of NET4/Tmem53 were dependent upon active p38 MAP kinase. The finding that roughly a fifth of nuclear envelope transmembrane proteins screened yielded alterations in flow cytometry cell cycle/DNA content profiles suggests a much greater influence of the nuclear envelope on the cell cycle than is widely held.

## Introduction

Several proteins of the nuclear envelope are linked to human diseases ranging from muscular dystrophies to neuropathy, bone diseases, and progeroid aging syndromes [1], [2]. These proteins include the intermediate filament A/C Lamins and several proteins integral to the nuclear membrane. Favored molecular mechanisms to explain how mutations in nuclear envelope proteins produce pathology include loss of nuclear mechanical stability, alterations in gene expression, and cell cycle/stem cell maintenance defects (reviewed in [2], [3], [4]). However, the known functions of the proteins mutated in disease are insufficient to fully explain the pathologies observed without assistance from partner proteins that thus far have not been identified.

The first indication of a link between nuclear envelope diseases and the cell cycle came from studies with specific mutations in the nuclear envelope transmembrane protein (NET) Emerin linked to Emery-Dreifuss muscular dystrophy. It was reported that two disease-linked mutations prolonged S-phase from 12 h to 22 h when overexpressed in COS-7 cells [5]; however similar effects were not observed in all disease mutants and so this was not investigated in further detail. In *C. elegans* disruption of Emerin alone did not have a strong effect on the cell cycle, but when combined with disruption of a second NET, MAN1, it did [6]. Loss of Emerin has also been reported to interfere with retinoblastoma protein (pRb)-regulated genes in mouse and consequently with myogenic differentiation [7], and the same pRb-dependent cell cycle exit is disrupted in nuclear envelope-linked muscular dystrophy [8]. pRb is a tumor suppressor that regulates the cell cycle at the G1/S transition by regulating the E2F family of transcription factors (reviewed in [9]). pRb also interacts with Lamin A [10], but this is thought to principally involve the nucleoplasmic and not the nuclear envelope pool of Lamin A because it operates in a complex with LAP2α, a soluble splice variant of the nuclear envelope protein LAP2 that is principally found in the nucleoplasm [11], [12], [13].

To determine if any of several newly identified nuclear envelope proteins play a role in the cell cycle, 39 novel confirmed NETs were screened for their ability to alter flow cytometry cell cycle/DNA content profiles when exogenously expressed. These NETs were identified in two recent proteomic analyses of liver and blood cells [14], [15]. Seven of the NETs tested showed an increase in the 4N∶2N ratio while one showed a decrease. To determine if pathways affected by these NETs involved the p53 master cell cycle regulator, these eight NETs were retested in p53^−/−^ cells. The change in 4N∶2N ratios still occurred in the absence of p53 for most NETs, but the effect of NET4/Tmem53 and NET59/Ncln was lost. NET4/Tmem53 was selected for a more detailed analysis of how it interacts with the p53 pathway. Knockdown of NET4/TMEM53 resulted in cell cycle withdrawal, apparently through activation of the p38 kinase with consequent upregulation of p53 and p21 and downregulation of phosphorylated pRb.

## Results

### A screen for NETs that alter flow cytometry profiles

To identify nuclear envelope proteins that might contribute to cell cycle progression, a collection of 39 NETs were screened for their ability to affect flow cytometry cell cycle/DNA content profiles. All NETs were fused to a monomeric red fluorescent protein (mRFP) tag at their carboxyl-termini and were previously confirmed to target to the nuclear envelope [14], [15], [16]. HEK293T human embryonic kidney cells were used for the screen because this cell line is efficiently transfected, easily recovered from plates for the flow cytometry experiments, and has a relatively stable karyotype compared to other commonly used lines such as HeLa, U2OS or HT1080 cells. Tagged NETs were transiently transfected into the HEK293T cells and after 40–48 h of expression the frequency of live cells with 2N or 4N DNA content was measured by flow cytometry.

DNA profiles were acquired for both the transfected cells (mRFP positive) and the untransfected population for each transfection. Thus the use of transient transfections provided an internal control for each experiment that removed any cell cycle variation between plates and/or due to the transfection reagent. For each NET at least three independent flow cytometry experiments were performed, each on different days and with a minimum of 1,000 singlet transfected cells (and in most cases >5,000 cells) analyzed. For those NETs where a strong effect was observed, additional repeats were done with 20,000 transfected cells analyzed to increase confidence.

**Figure 1:**
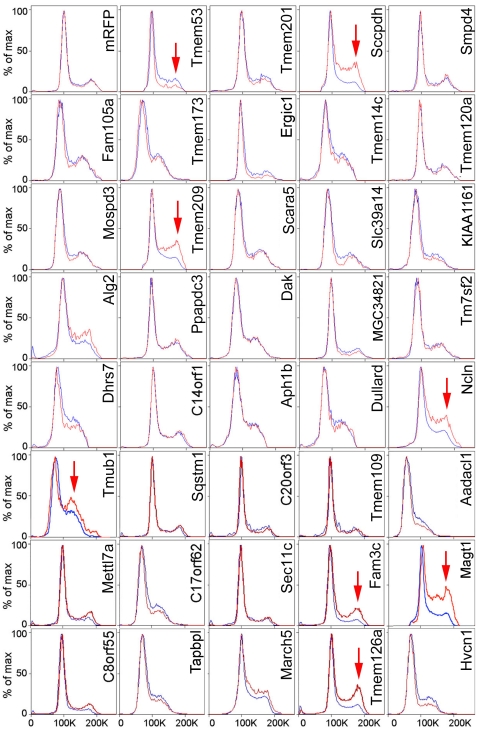
Changes in flow cytometry cell cycle profiles for cells overexpressing NETs. HEK293T cells expressing mRFP-NET fusions were recovered by trypsinization and analyzed by flow cytometry at 48 h post-transfection. Data were analyzed using FlowJo software and histogram overlays are displayed as %Max, scaling each curve to mode = 100%. The red line is the mRFP expressing cells in the population while the blue line is the untransfected cells in the population (the majority of cells were not transfected). The transfected and untransfected populations were both set on the scale to 100 for the 2N population so that increases or decreases in the 4N peak reveal changes in the cell distribution. Arrows indicate significant changes in cell cycle profile between transfected and non-transfected cells.

Examples of flow cytometry cell cycle/DNA content profiles are shown in Figure 1 with the untransfected cell traces (blue) overlaid with those of the mRFP-expressing population (red). Cell fragments and apoptosing cells were excluded based on propidium iodide (PI) staining and FSC/SSC (from light scattering). The flow cytometry profiles for the mRFP control and many other NETs tested were indistinguishable from those of untransfected cells in the same population or only exhibited minor differences. By contrast, NET11/Sccpdh, NET31/Tmem209, NET59/Ncln, Tmub1, Fam3c, Magt1 and Tmem126a all yielded striking accumulations of cells with a 4N DNA content suggesting an increased G2/M population (Figure 1). NET4/Tmem53 yielded a different effect, exhibiting a reduction in cells with 4N DNA content suggestive of more cells in the G1 phase of the cell cycle. The percentages of cells in G1, S, and G2/M phases based on DNA content are listed in Table 1. While effects of these eight NETs were the most striking and reproducible, one cannot discount that some NETs that caused minor changes might also be relevant to the cell cycle.

**Table 1:** Percentage of cells in each cell cycle phase by flow cytometry upon exogenous expression of NET-mRFP fusions.

NET	mRFP negative			mRFP positive		
	% G1	% S	% G2/M	% G1	% S	% G2/M
untransfected	60	14	21	-	-	-
mRFP	61	14	20	62	13	20
NET4/Tmem53	62	14	20	74	8	14
NET11/Sccpdh	60	14	20	37	18	38
NET31/Tmem209	58	15	21	39	15	38
NET51/C14orf1	62	16	18	60	17	19
NET59/Ncln	59	15	22	37	19	35
Fam3c	62	10	18	44	10	30
Magt1	65	12	19	53	14	26
Tmub1	63	13	19	50	14	31
Tmem126a	65	13	14	53	17	26

**Figure 2:**
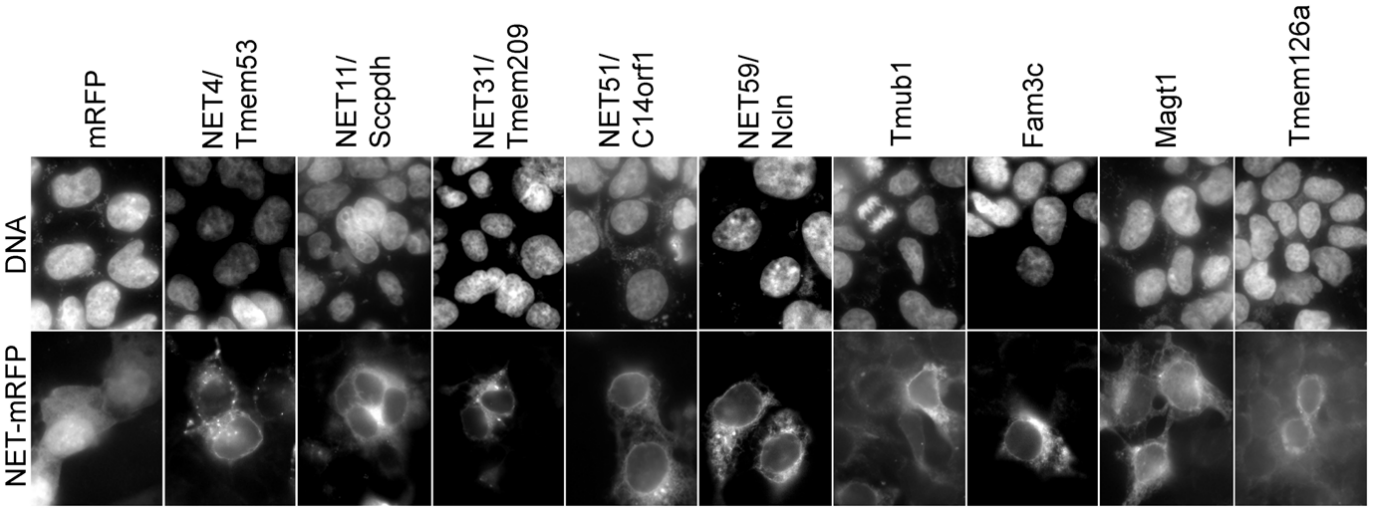
The changes in flow cytometry profile of cells expressing NETs are not caused by aberrant nuclear morphology of cells. Images of HEK293T cells expressing NET-mRFP shows nuclear morphology depicted by DAPI staining (upper panel) compared to mRFP signal (lower panel) indicating cells expressing NETs.

It is possible that some NETs positive in the screen could have altered flow cytometry DNA content profiles because of aberrant nuclear morphologies as opposed to effects on cell cycle progression (though this could in turn reflect problems with cytokinesis). To test if this was a likely explanation, cells from the transfected populations were imaged by fluorescence microscopy. Representative images revealed no gross aberration in nuclear morphology within the transfected population, indicating this is unlikely to have affected the flow cytometry results (Figure 2). Nuclear envelope targeting is not always extremely clear in the HEK293T cells either because NETs have multiple localizations or because the high expression saturates binding sites at the nuclear envelope in these cells; however, all NETs tested here were previously confirmed to target to the nuclear envelope [14], [15], [16].

### Some NETs depend on p53 and/or pRb for their effects

**Figure 3:**
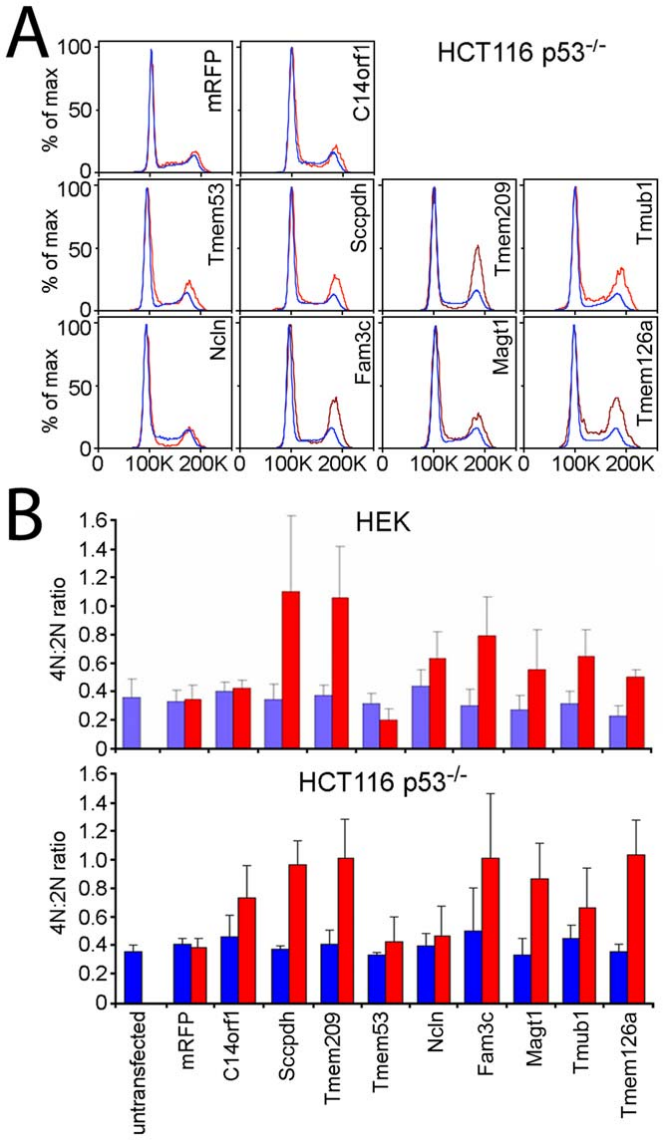
Cell cycle effects of NET4/Tmem53 and NET59/Ncln depend on p53. (A) Flow cytometry profiles of cells expressing NET-mRFP fusions in HCT116 p53−/− cells. Most NETs that had produced increases in the 4N population in Figure 1 yielded similar increases in the 4N population in HCT116 p53−/− cells; however, NET4/Tmem53 and NET59/Ncln lost their effects. (B) The percentage of cells in the 4N and 2N populations were calculated and 4N∶2N ratios were plotted from at least three separate experiments with standard errors shown. The results for the HEK293T cells are shown above those for the HCT116 p53−/− cells.

The cell cycle protein p53 is often referred to as a master regulator because it has a role in a large number of cell cycle pathways. The ability of the eight NETs identified in the initial screen to alter the flow cytometry DNA content profiles was re-examined in the HCT116 p53^−/−^ cell line. The controls mRFP and NET51/C14orf1 had not yielded changes in the flow cytometry DNA content profile in the HEK293T cells and similarly yielded no significant changes in the p53^−/−^ cells (Figure 3A). Correspondingly, NET11/Sccpdh, NET31/Tmem209, Tmub1, Fam3c, Magt1, and Tmem126a that had exhibited increases in the 4N population in the HEK293T cells yielded similar changes in the p53^−/−^ cells; so p53 does not appear to be involved in the potential effects of these NETs on the cell cycle. By contrast, in the p53^−/−^ cells NET59/Ncln no longer exhibited a 4N increase and NET4/Tmem53 no longer exhibited a 4N decrease as had been observed in the HEK293T cells. Thus changes in the flow cytometry profiles from expression of NET59/Ncln and NET4/Tmem53 appear to be p53 dependent. To better compare the results in the p53 positive and negative cells, the 4N∶2N ratios from both cell lines were plotted for this set of NETs (Figure 3B). For NETs other than the two that lost their effects a similar pattern was observed between the two cell lines, although in some cases the 4N∶2N ratio increase was slightly higher in the HCT116 p53^−/−^ cell line. NET4/Tmem53, a previously uncharacterized protein with no known functional domains, was subsequently followed in more detail.

**Figure 4:**
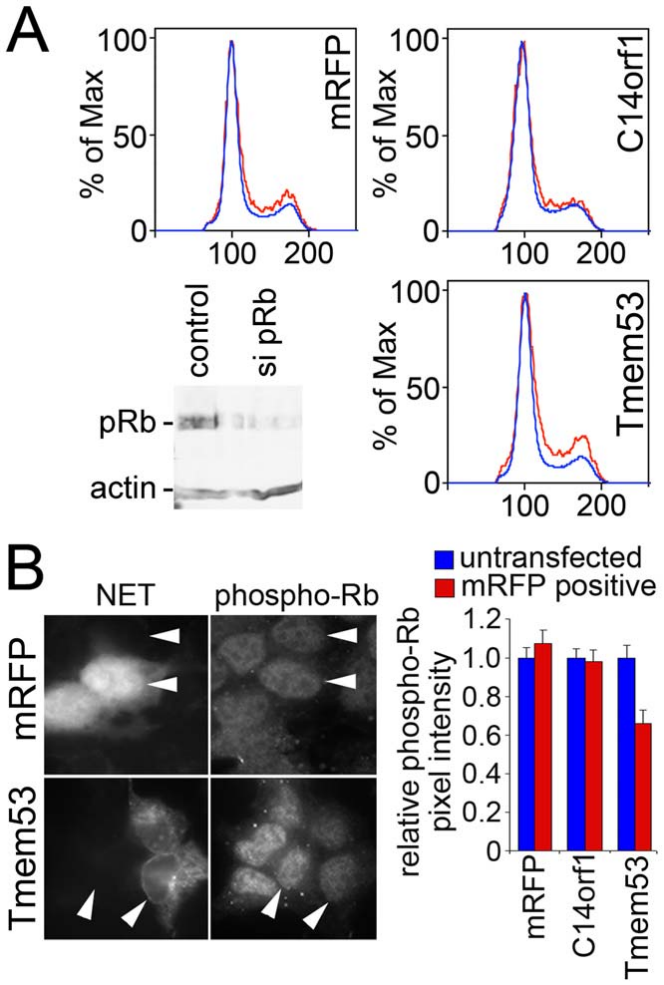
pRb is also involved in NET4/Tmem53 effects. (A) pRb was knocked down by siRNA in HEK293T cells subsequently transfected with NETs. Cell cycle profiles are shown for these cells expressing NET4/Tmem53 or controls of mRFP alone or NET51/C14orf1 that had no effect in the original flow cytometry screen. The 2N accumulation effect of NET4/Tmem53 on the cell cycle profile observed in HEK293T cells was lost when pRb levels were reduced. The knockdown of pRb is confirmed in the lower left corner. (B) Photomicrographs of cells overexpressing the RFP alone control or NET4/Tmem53. Cells were stained with an antibody that recognizes pRb phosphorylated on serine 780. NET/mRFP signal is shown in the left panels to identify transfected cells and phospho-pRb staining in the right panels. In each panel two adjacent cells are marked by arrowheads, one transfected and the other not transfected. All micrographs were taken at the same exposure time. The graph on the right shows quantification of the pixel intensity for the phospho-pRb staining. The pixel intensity for untransfected controls internal to each micrograph was set to 1 and average relative values from 40 transfected cells and 40 untransfected cells for each transfection are shown with standard errors.

Cell cycle effects dependent on p53 often involve changes in pRb [17] and links have previously been identified between pRb and nuclear envelope-associated proteins [10], [11], [12], [13]. To determine if pRb also plays a role in the NET4/Tmem53-directed effects, flow cytometry cell cycle/DNA content profiles were determined for cells expressing NET4/Tmem53 or controls in HEK293T cells with normal or reduced levels of pRb, using siRNA oligos to knock down pRb. The reduction in the 4N∶2N ratio caused by NET4/Tmem53 was lost in the pRb-depleted cells (Figure 4A). As pRb phosphorylation is crucial for its role in cell cycle progression [18], [19], [20], antibodies to a form of pRb phosphorylated at serine 780 were utilized to test if phosphorylated pRb levels were affected in the NET4/Tmem53 transfected cells. Because of low and variable transfection efficiencies this could not be assayed at a population level; thus transfected cells were stained with the antibodies and the levels of the phosphorylated pRb in the nucleoplasm were quantified by measuring the average pixel intensity (Figure 4B). Plotting these values revealed a significant loss of phosphorylated pRb in cells expressing NET4/Tmem53.

### RNAi knockdown of NET4/Tmem53

Further study of the pathways through which NET4/Tmem53 affects cell cycle regulation would not be practical using exogenous expression because transfection efficiencies were too low (5–10%) to be able to quantify changes in pathway components by Western blot. The p53, p38 and p21 antibodies used in subsequent assays were tested by immunocytochemistry, but proved inadequate for quantification due both to a diffuse distribution throughout the cell body and cell-to-cell variation in intensities that appears to result from induction of stress pathways in some cells during transfection (data not shown). Therefore, to further elucidate the pathways through which NET4/Tmem53 affects the cell cycle, its knockdown was attempted.

**Figure 5:**
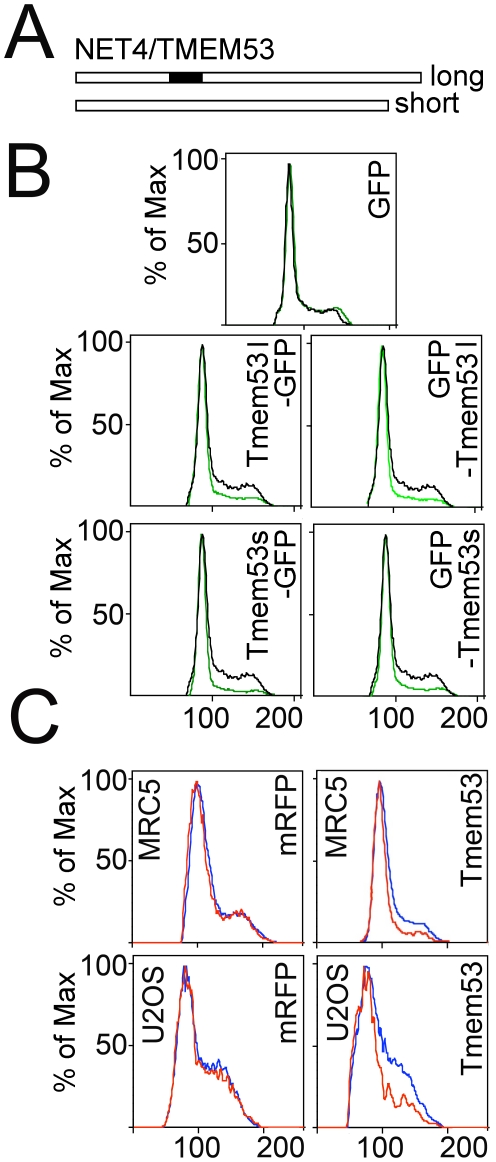
Splice variants of NET4/TMEM53 and the effect of knockdown in U2OS and MRC5 cells. (A) Two splice variants of NET4/TMEM53 were cloned that differed only by a 33 amino acid insertion roughly a third of the way into the protein. (B) Both long (l) and short (s) splice variants had the same effect on cell cycle/DNA content profiles and effects were independent of whether the GFP tag was on the amino terminus or carboxyl terminus. (C) Because siRNAs failed to knock down NET4/TMEM53 in HEK293T cells, the cell cycle/DNA content profile effect of the original NET4/TMEM53-mRFP construct was tested on U2OS and MRC5 cells as alternatives for knockdowns.

There are both long and short splice variants of NET4/TMEM53 (Figure 5A). To determine whether it would be better to design siRNA oligos to knock down one or both (the original screen used the shorter variant), long and short splice variants of NET4/TMEM53 with the GFP moiety at either terminus were cloned and then tested to determine if both could produce the flow cytometry cell cycle/DNA content ratio effect of the original mRFP construct. All constructs yielded the same effect (Figure 5B). Multiple siRNA oligos were then generated that should knock down both long and short splice variants. Although the HEK293T cells were ideal for the flow cytometry-based screen due to their generally high transfection efficiencies and comparatively stable DNA content, for subsequent more directed cell cycle experiments MRC5 primary fibroblasts [21] and the U2OS osteosarcoma cell line were used because they have comparatively more operational checkpoint machinery. Moreover, the primary fibroblasts can enter a state of senescence that is not possible for HEK293T cells. Before proceeding, the ability of NET4/Tmem53-mRFP expression to alter flow cytometry DNA content profiles was evaluated in both MRC5 primary fibroblasts and the U2OS cells, confirming that a comparable effect to that observed in HEK293T cells occurred in the MRC5 and U2OS cells (Figure 5C).

**Figure 6:**
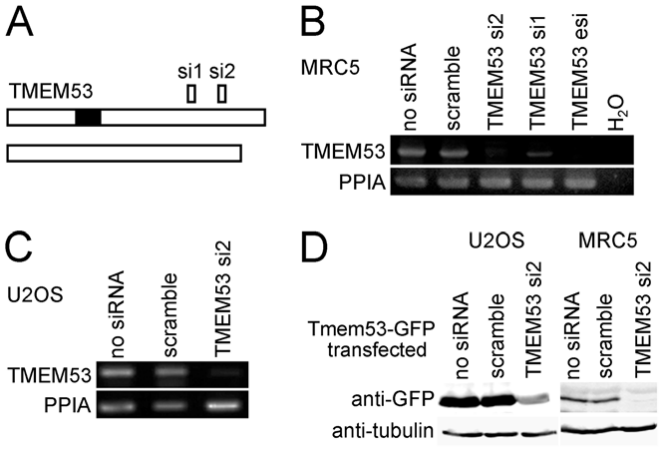
Knockdown of NET4/TMEM53 in U2OS and MRC5 cells. (A) Splice variants and position of knockdown oligos. (B) NET4/TMEM53 transcript levels were effectively knocked down in MRC5 cells using both siRNA oligos and an esiRNA. Peptidylprolyl isomerase A (PPIA) was used as a control for normalization. (C) NET4/TMEM53 transcript levels were also knocked down in the U2OS cell line using the siRNA oligo TMEM53 si2. The scramble siRNA oligo had no effect. PPIA was used as a control for normalization. (D) Western blot demonstrating the knockdown of NET4/Tmem53. Because antibodies to NET4/Tmem53 recognized multiple bands on Western blot at the expected molecular weight, knockdown of the protein was tested using a NET4/Tmem53-GFP fusion and GFP antibodies. The siRNAs were transfected 24 h after transfection of NET4/Tmem53-GFP in order to enable protein to be generated from the plasmid prior to the beginning of knockdown.

Two siRNA oligos (si1 and si2) that should each in theory knock down both long and short splice variants (Figure 6A) effectively knocked down NET4/TMEM53 transcripts in MRC5 cells (Figure 6B). The si2 was slightly more effective than the si1 and therefore initially used in preference, though all relevant findings were subsequently verified with both oligos. As *NET4/TMEM53* is an uncharacterized gene with no direct evidence for its full range of splicing possibilities, it is possible that additional splice variants exist that the two siRNA oligos do not knock down. Therefore, an esiRNA that should knock down any and all possible splice variants of NET4/Tmem53 was also tested and found to be effective in reducing NET4/TMEM53 transcripts in MRC5 cells (Figure 6B). Knockdown of NET4/TMEM53 transcripts was also successful in U2OS cells (Figure 6C).

Available NET4/Tmem53 antibodies could not be used to determine if the NET4/Tmem53 protein was also being knocked down because they recognized several closely migrating bands where NET4/Tmem53 protein is expected to migrate on Western blot. Nonetheless, the endogenous protein is very likely to be reduced because cells expressing NET4/Tmem53-GFP prior to addition of siRNA oligos for NET4/TMEM53 knockdown exhibited a loss of the fusion protein with both GFP and NET4/Tmem53 antibodies (Figure 6D and data not shown). This indirect approach typically indicates knockdown of the endogenous protein [22].

The effect of NET4/TMEM53 knockdown on flow cytometry cell cycle/DNA content profiles was next determined. An increase was observed in the population of cells with a 2N amount of DNA that was similar to the increase resulting from exogenous NET4/Tmem53 expression (Tables 1 and 2). A corresponding reduction was observed in the population of cells with 4N DNA content both with the knockdown and with overexpression. Both MRC5 and U2OS cell lines were subsequently used in parallel for most experiments to ascertain if any observed effects required the more reliable cell cycle checkpoints of primary cells (MRC5) and thus were not recapitulated in transformed cell lines such as the U2OS line that has stable p53- and pRb-dependent checkpoints but is defective for p16INK [23], [24].

**Table 2:** Percentage of cells in each cell cycle phase by flow cytometry for knockdown of NET4/TMEM53 and controls in U2OS cells.

	%G1	%S	%G2/M
untransfected	58	10	32
scramble	59	11	30
oligo si 2	65	12	22

### NET4/TMEM53 knockdown causes premature senescence in primary fibroblasts but only a cell cycle delay in the transformed U2OS cells

**Figure 7:**
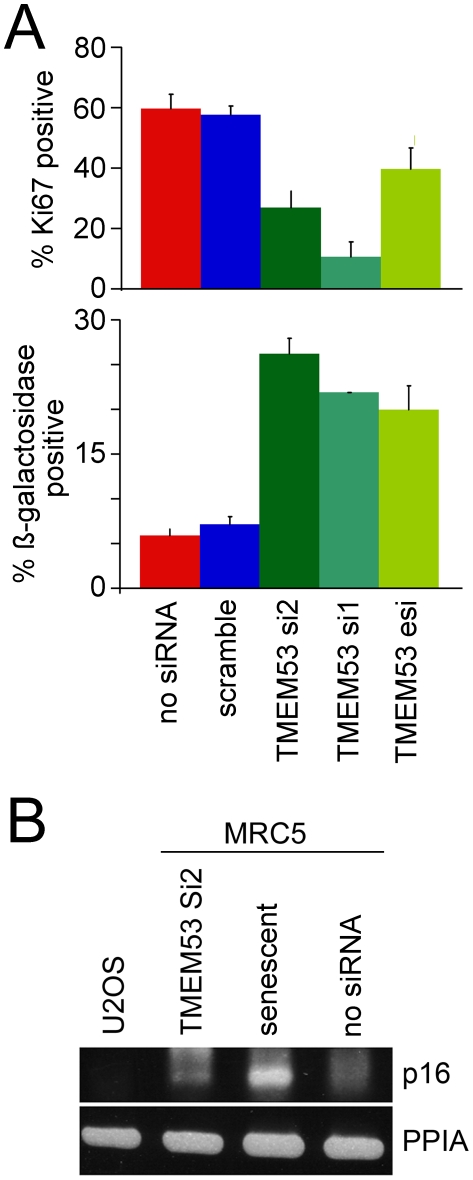
NET4/TMEM53 knockdown results in cell cycle withdrawal. (A) NET4/TMEM53 knockdown with both siRNAs and the esiRNA all resulted in a reduced number of actively proliferating cells as measured by Ki-67 staining (upper graph) and an increase in senescent cells as measured by ß-galactosidase staining (lower graph). The results from three separate experiments with standard errors are shown. (B) Differences between MRC5 and U2OS cells in NET4/Tmem53-induced senescence are likely due to the absence of p16INK in U2OS cells. RT-PCR was used to detect transcript levels of p16INK. In U2OS cells no transcripts were observed while in senescent MRC5 cells transcript levels were greatly increased compared to cycling untransfected MRC5 cells. Knockdown of NET4/Tmem53 also induced p16INK expression in MRC5 cells, albeit less so than by full senescence.

When MRC-5 cells were transfected with siRNA oligos or esiRNA for NET4/TMEM53 the number of cells per dish appeared to be lower than in cultures transfected with the scrambled control siRNA oligo, yet there did not seem to be an increase in apoptotic cells. In order to establish the cause of this difference, cell proliferation was assessed using antibodies to the nuclear protein Ki-67 that is present only in proliferating cells [25], [26]. In cells knocked down for NET4/TMEM53 (Figure 7A, upper graph) there was a notable decrease in the frequency of Ki-67 positive cells from 57.6% in the control scrambled oligo transfected cells to 26.9% for the NET4/TMEM53 si2 transfected cells and 10.9% for the NET4/TMEM53 si1 transfected cells. The esiRNA also confirmed the phenotype (39.6% Ki-67 positive cells), further indicating its specificity to the NET4/Tmem53 knockdown.

The marked reduction in cell proliferation upon NET4/TMEM53 knockdown could indicate a permanent arrest or a temporary arrest from which cells could subsequently recover. ß-galactosidase activity, a characteristic feature of senescent cells [27], was therefore assessed in MRC5 primary fibroblasts knocked down for NET4/TMEM53. At 72 h post transfection, the percentage of ß-galactosidase positive cells was ∼3 times higher in cell cultures transfected with NET4/TMEM53 siRNA oligos or esiRNA as compared to untransfected cultures or those transfected with the control scramble oligo (Figure 7A, lower graph). This suggests a tendency of NET4/Tmem53 deficient MRC-5 cells to withdraw from the cell cycle due to premature senescence, which is a programmed cell response to many extra- and intracellular stresses with features similar to proliferative senescence and leading to permanent cell cycle exit.

By contrast, when NET4/TMEM53 siRNA oligo si2 was transfected into the transformed osteosarcoma U2OS cell line no decrease of Ki-67 expression or increase of ß-galactosidase was detected (data not shown), indicating that these cells do not fully exit the cell cycle. This difference is likely due to the absence in U2OS cells of p16INK, as this kinase has been reported to be important for establishment of senescence [23], [24]. Confirming these reports, transcript levels of p16INK were undetectable in U2OS cells and strongly upregulated in senescent MRC5 cells (Figure 7B). Knockdown of NET4/TMEM53 resulted in a small increase in p16INK transcript levels (Figure 7B), consistent with the hypothesis that its absence in U2OS cells underlies the failure to become senescent. Despite the inability to senesce, some effects on the cell cycle still occurred in U2OS cells knocked down for NET4/TMEM53 as BrdU incorporation was reduced by nearly 50% (data not shown), indicating that the frequency of cells in S phase was greatly reduced. This could indicate that these cells spend an extended period in G1, consistent with the flow cytometry data.

### NET4/TMEM53 knockdown results in alteration of levels of several cell cycle regulators

**Figure 8:**
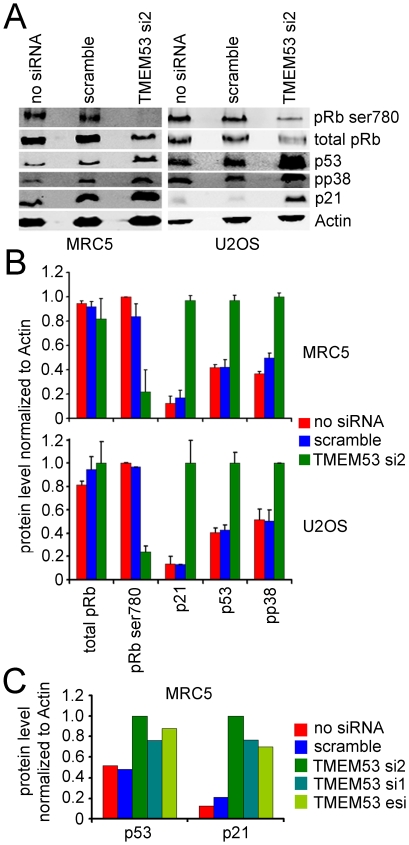
NET4/TMEM53 knockdown alters levels of several cell cycle proteins. Protein lysates were recovered from both MRC5 and U2OS cells that were either untransfected, transfected with a scrambled siRNA, or transfected with the NET4/TMEM53 si2 oligo. (A) Western blots reacted with antibodies to pRb phosphorylated on serine 780, total pRb, p53, phosphorylated p38MAP kinase, p21, and actin as a loading control. Note that some variation in total pRb levels was observed between experiments, causing the ratio of phosphorylated pRb to total pRb to be also somewhat variable. Because the phosphorylated form migrates slightly differently on SDS-PAGE the change in the spread of the band gives a greater appearance of such variability than is actually measured upon quantification. (B) Quantification of levels of each protein normalized to actin using a LI-COR Odyssey and fluorescent secondary antibodies. Averages from three experiments are shown with standard errors. (C) To ensure that results reflected effects of NET4/TMEM53 knockdown and not off-target effects both siRNA oligos and the esiRNA were tested for effects on p53 and p21 levels. Similar results were observed with all NET4/TMEM53 knockdowns.

To further understand the pathways mediating the effect of NET4/Tmem53 on the cell cycle, levels of various cell cycle regulators before and after NET4/Tmem53 depletion were quantified by Western blot. In both MRC5 and U2OS cells the p53 protein level was increased ∼2-fold upon loss of NET4/Tmem53 while p21 levels increased ∼7-fold (Figure 8A,B). Total levels of pRb remained relatively unchanged, but pRb became hypophosphorylated when NET4/Tmem53 was knocked down and phosphorylated p38MAP kinase levels increased (Figure 8A,B). It should be noted that although total pRb levels were unchanged on average over several experiments with NET4/TMEM53 knockdown, there was reasonable variability in the levels of total pRb among experiments. Thus the ratio of phosphorylated pRb to total pRb was likely also somewhat variable, but levels of the phosphorylated pRb were notably reduced in all experiments. To confirm that the effect observed was specific for NET4/TMEM53 knockdown the experiment was repeated using oligos si1, si2, and the esiRNA. The same result was observed with p53 and p21 increasing upon loss of NET4/Tmem53 (Figure 8C).

**Figure 9:**
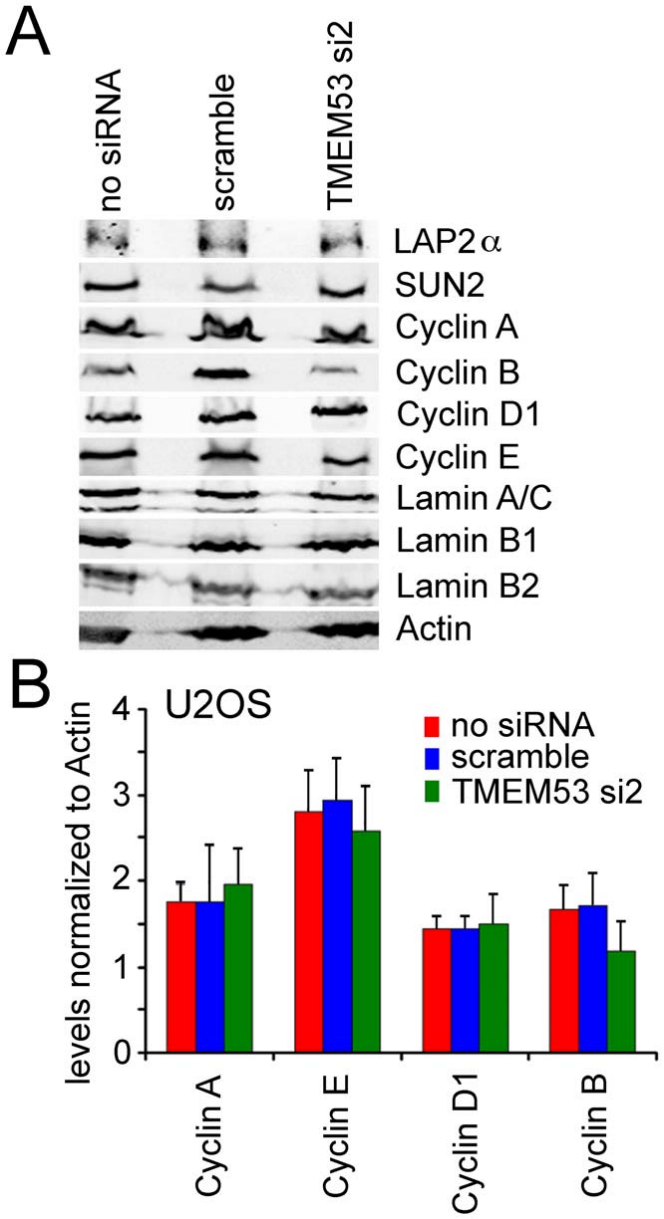
NET4/TMEM53 knockdown did not affect levels of other nuclear envelope proteins linked to the cell cycle or cyclins. (A) Protein lysates were recovered from U2OS cells treated as in Figure 8 and reacted on Western blots with antibodies to the various proteins. This experiment was repeated 3 times and a representative Western blot is shown. (B) Cyclin levels were quantified from three separate experiments analyzed by LI-COR using fluorescent secondary antibodies and are plotted normalized to the actin control. Standard errors are shown.

By contrast no changes were observed in levels for other NE proteins previously shown to contribute to cell cycle regulation Emerin, LAP2 (both soluble and transmembrane splice variants alpha and beta), or Lamin A (Figure 9A). Neither were differences observed in cyclins A, C, D, or E (Figure 9A,B). Cyclin B levels may have been slightly reduced; however, considerable variability was observed between all experiments and so no clear conclusion could be drawn (data not shown).

### NET4/Tmem53 effects on cell cycle protein levels are mediated by the p38MAP kinase

The p38MAP kinase that was upregulated by NET4/TMEM53 knockdown mediates cell response to range of stresses, such as UV irradiation, osmotic shock, heat shock, starvation and cytokines [28]. p38 phosphorylates and stabilizes p53, which activates transcription of the cdk inhibitor p21 that subsequently blocks the cell cycle [17]. As this study has shown that p21 is also upregulated by NET4/TMEM53 knockdown, it seemed likely that the effects on cell cycle proteins p53, pRb and p21 are mediated by the p38MAP kinase.

**Figure 10:**
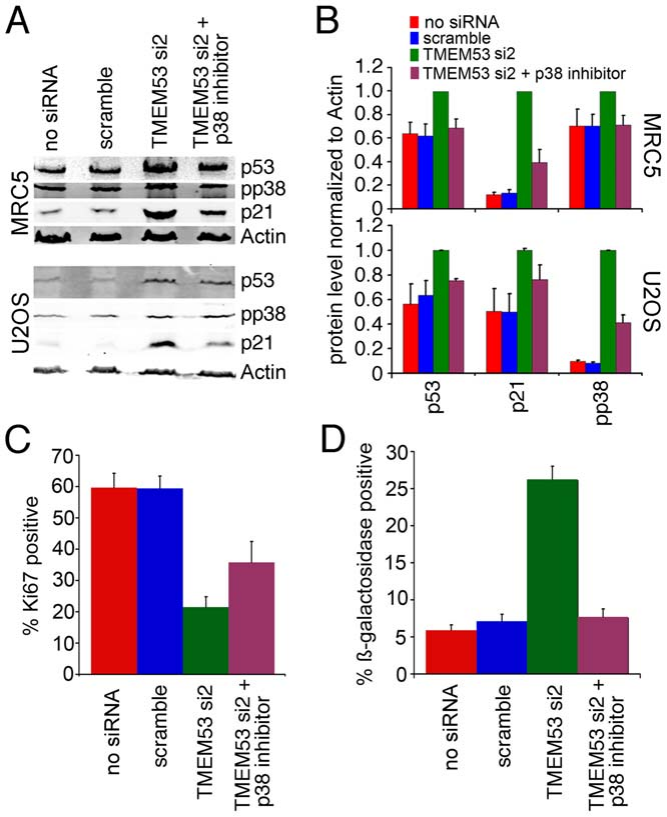
p38MAP kinase mediates the NET4/Tmem53 effects on cell cycle proteins. MRC5 and U2OS cells transfected with either siRNA oligos or controls were treated 24 h after transfection with a specific inhibitor of the p38MAP kinase. (A) Protein lysates were generated after a further 48 h and analyzed by Western blot for levels of p53, phosphorylated p38, and p21. This experiment was repeated 3 times and a representative Western blot is shown. (B) Levels of p53, p21 and phosphorylated p38 were quantified from three separate experiments analyzed by LI-COR using fluorescent secondary antibodies and are plotted normalized to the actin control with standard errors. In all cases the p38MAP kinase inhibitor blocked the effects of NET4/TMEM53 knockdown on levels of these cell cycle proteins. (C) The change in the percentage of Ki-67 positive cells induced by NET4/TMEM53 knockdown is partially abrogated by blocking the function of p38 kinase. (D) Effects of NET4/TMEM53 knockdown on senescence, as determined by ß-galactosidase staining, are abrogated by blocking p38MAP kinase function.

To further test if the increases in p53 and p21 were dependent on p38MAP kinase, a specific p38 inhibitor SB203580 (Calbiochem) was added to the cultures 24 h after transfection with the siRNA oligos. The cells were harvested 48 h later and levels of p53, p21, and phosphorylated p38 measured by Western blot. In all cases the increases resulting from knockdown of NET4/TMEM53 were lost when p38MAP kinase was inhibited (Figure 10A,B). Moreover, the NET4/TMEM53 knockdown-mediated decrease in Ki-67 positive cells was partially abrogated and the increase in ß-galactosidase staining cells was completely abrogated (Figure 10C,D). Thus, p38MAP kinase appears to be activated by NET4/TMEM53 knockdown and mediates most of the NET4/Tmem53 effects on the cell cycle.

## Discussion

This flow cytometry-based screen identified several NETs with likely cell cycle functions. These NETs are generally uncharacterized proteins, many of which have conserved domains of unknown function. However, NET59/Ncln has separately been linked to a pathway that could intersect with cell cycle regulation: this NET interacts with Nomo1 that binds Smad proteins and thus intersects with TGFß signaling pathways [29]. The NET MAN1 has also been found to interact with Smad proteins [30], [31] while the NET Emerin interacts with ß-catenin [32] and disruption of these two NETs yields cell cycle defects [6]. Thus, combined signaling/cell cycle effects appear to be a common trait among NETs and it is likely that further analysis of the other NETs identified in this screen will yield some similar intersections of nuclear envelope proteins with cell cycle pathways. That these 8 NETs represent roughly 20% of the proteins tested in the flow cytometry cell cycle/DNA content screen and that several recent studies have found NETs associated with centrosomes or the mitotic spindle [33], [34], [35] suggest that a great many nuclear envelope proteins will be found to contribute to cell cycle regulation.

Here, additionally, NET4/Tmem53 has been demonstrated to influence cell cycle regulation, affecting maintenance of the proliferative state of cells. Although the mechanism by which NET4/Tmem53 expression levels activate p38MAP kinase has thus far been elusive, it is clear that the effect of NET4/Tmem53 on proliferation is through p38MAP kinase-dependent upregulation of p53 and p21 with subsequent reduction in levels of phosphorylated pRb. Thus unlike Lamin A and LAP2α that exert effects directly on pRb to influence cell proliferation [11], [12], [13], NET4/Tmem53 acts upstream through stress signaling pathways.

NET4/Tmem53 was originally identified in a proteomic analysis of liver nuclear envelopes [14] and high-resolution microscopy on tagged exogenously expressed protein suggests that it resides in the outer nuclear membrane [16]. Antibodies generated to NET4/Tmem53 were previously shown to principally stain the nuclear periphery in liver tissue cryosections and nuclear envelope versus microsomal fractions isolated from rodent liver [16]; however, despite reactivity on tissue sections, these antibodies recognize several additional bands at the expected molecular weight of NET4/Tmem53 in cultured cells and so could not be exploited in the current study. The indication of NET4/Tmem53 as an outer nuclear membrane protein is not inconsistent with NE functions on the cell cycle. For example, Emerin, originally thought to reside only in the inner nuclear membrane [36], affects signaling pathways and has recently been shown to function also in the outer nuclear membrane and ER [37].

A potential functional relevance for NET4/Tmem53-induced cell cycle regulation is suggested by observations that NET4/TMEM53 is highly expressed in liver cells and adipocytes according to the BioGPS transcriptome database [38], [39]. Recent studies have shown that the differentiation of mesenchymal stem cells (MSCs) is influenced by mechanical strain. In response to the application of physical strain MSCs are prevented from differentiating along an adipogenic lineage and instead are redirected towards an osteoblast lineage [40], This gives rise to the possibility the physical stress provides a homeostatic mechanism for mesenchymal lineage selection. Since it has already been shown the NE plays a key role in propagating stress signaling and sensing [41], it is important to understand which NE proteins interact with stress signaling pathways and how they might influence stem cell self renewal and differentiation. We believe that our data suggests that NET4/Tmem53 has such a role. Future studies will focus on putative regulation of adipogenic stem cells by NET4 and its possible involvement in Laminopathies such as Dunnigan-type familial partial lipodystrophy (FPLD; [42], [43]) or mandibuloacral dysplasia (MADA; [44]).

## Methods

### Plasmid construction

NET expressing plasmids were generated from IMAGE clones as previously described [14], [15], [16]. All were under regulation of the CMV promoter with their carboxyl-termini fused to monomeric red fluorescent protein (mRFP).

### Cell culture and transient transfection

HEK293T (from ATCC; [45]), U2OS (from ATCC; [24]), MRC5 (from the EUACC; [21]) and HCT116 p53^−/−^ (kind gift of B. Vogelstein, Johns Hopkins University; [46]) cells were maintained in high glucose DMEM (Invitrogen) supplemented with 10% fetal calf serum, 100 units/ml penicillin and 100 µg/ml streptomycin sulfate (Invitrogen). For U2OS siRNA assays cells were seeded at 30% confluency and the next day transfected using Dharmafect transfection reagent 1 according to the manufacturer recommendations (Dharmacon RNA Technologies). MRC5 cells were trypsinized at ∼80% confluency and 3×10^6^ cells were aliquoted for nucleofection using Kit R, Program V-020 (Lonza). The cells were transfected with either 8 µg of siRNA oligo or 12 µg of esiRNA (esiRNA human TMEM53, Sigma) and plated to 10 cm dishes with several coverslips. At 48 or 72 h coverslips were removed and fixed for immunocytochemistry and the remaining cells on the plate were harvested by trypsinization and lysed in sample buffer for SDS-PAGE and Western blotting. Alternatively they were lysed in Trizol reagent (Sigma) for isolation of RNA. HEK293T cells were either nucleofected using Kit V Program Q-001 (Lonza) or were transfected using Fugene 6 or HD (Roche). For microscopy, cells were generally plated on coverslips in 24 well dishes after nucleofection or were first plated and then transfected using Fugene. In this case, plating was at ∼10% confluency so that cells did not reach confluency before 30 h post-transfection when they were fixed. Then, roughly 12 h after plating, DNA was transfected using Fugene. For p38MAP kinase inhibitor experiments the specific inhibitor SB203580 (Calbiochem) was added to cultures at 10 µM concentration 12 h after transfection with siRNA oligos.

### Cell cycle assay

Plasmids encoding different NETs fused to mRFP were transfected into HEK293T cells using Fugene 6 transfection reagent (Roche). At 48 h post-transfection, the DNA stain Hoechst 33342 (Molecular Probes) was added to the cells at a final concentration of 5 µg/ml and left to incubate at 37°C for a period of 30 min to 60 min. Cells were harvested by trypsinization, trypsin was inactivated with serum and cell pellets were collected by centrifugation at 250×g for 5 min at RT, washed once in PBS and resuspended in 1 ml of PBS. Cells were immediately analyzed on an LSR II flow cytometer (BD Bioscience, UK) equipped with 488 nm and 350 nm lasers and appropriate filters. Cells with fragmented DNA that might be undergoing necrosis or apoptosis and cell aggregates were excluded from analysis by application of electronic gates. Cell cycle analysis was carried out on the live singlets gate using *FlowJo* software (TreeStar, Inc). At least 10,000 cells were scored for the total live singlets and 1,000 cells for the mRFP positive live singlets. Each experiment was repeated at least 2 times for negative results and 3 times for those with a positive effect on the cell cycle. For further confirmation NETs that showed an effect on the cell cycle in 3 independent experiments (NET4/Tmem53, NET11/Sccpdh, NET31/Tmem209, NET59/Ncln, Tmub1, Fam3c, Magt1 and Tmem126a) were repeated a 4^th^ time where at least 20,000 mRFP positive intact singlets were counted. Data are displayed in the form of histogram overlays using %Max option, which scales each population curve to mode = 100%.

### Antibodies

Antibodies to the following proteins were used: Ki-67 (610968, BD Transduction Lab), total Rb (4H1 9309, Cell signaling), phospho-Rb (9307, Cell Signaling), p21 (556430, BD Transduction lab), p53 rabbit (9282, Cell signaling), p53 mouse (NCL-p53-DO1, Leica), p38 total (9212, Cell Signaling), active p38 (V3281 Anti-active MAPK Family Sampler, Promega), cyclin E mAb clone HE12 (32-1600, Invitrogen), cyclin A mAb clone Cy-A1 (4710, Sigma), cyclin D (2922, Cell Signaling), cyclin B (SC245, Santa Cruz), LAP2ß (06-1002, Millipore), Lap2α previously described in [47], Lamin A and B1 (3262 and 3931) previously described in [48]. All fluorophore-conjugated secondary antibodies used for immunofluorescence were minimally cross-reactive from donkey (Jackson ImmunoResearch) or goat (Molecular Probes). For Western blotting IR800 conjugated goat anti-rabbit antibodies (LI-COR Biosciences) were used.

### Immunofluorescence microscopy

Cells transfected with NETs were fixed for 7 min in 3.7% formaldehyde, permeabilized for 6 min in 0.1% Triton X-100, blocked with 3% BSA in PBS, and reacted for 40 min at RT with antibodies to Ki-67 or phospho-Rb. After washing, appropriate fluorophore-conjugated secondary antibodies were added for 30 min at RT and washed. Cells were also stained with Hoechst 33342 (Molecular Probes) to visualize nuclei and mounted in fluoromount G (EM Sciences). For ß-galactosidase assays, histochemical staining at pH 6.0 was performed as described in [27].

Images were obtained using a Nikon TE-2000 microscope equipped with a 1.45 NA 100× objective, Sedat quad filter set, PIFOC Z-axis focus drive (Physik Instruments), and CoolSnapHQ High Speed Monochrome CCD camera (Photometrics) run by IPLab image acquisition software. Micrographs were saved from source programs as .tif files and prepared for figures using Photoshop 8.0.

Quantification of grey levels for NETs was performed using .tif images from IPLab imported into Image Pro Plus. Cells fixed and stained with phosho-Rb antibodies were imaged using identical settings. Average grey scale values (pixel) intensity were measured for at least 40 mRFP positive cells and as many untransfected cells on the same coverslips.

### Quantitative Western blotting

Cells were scraped and lysed in 50 mM Tris-HCl (pH 7.4), 150 mM NaCl, 2 mM MgCl_2_, 0.2% NP-40 in the presence of protease inhibitor cocktail (Roche 11 873 580 001) by heating at 65°C for 2 min and sonication in a sonibath at 4°C. Protein concentrations were determined using the Bradford Method (BioRad). An equal volume of protein sample buffer (100 mM Tris pH 6.8, 4 M Urea, 2% SDS, 50 mM DTT and 15% sucrose) was added and the samples were boiled at 100°C for 5 min then sonicated in a sonibath with high frequency for 10 min with 30 sec interval on/off. Equal amounts of protein were resolved by SDS-PAGE and transferred to Nitrocellulose membrane (LI-COR Biosciences). Membranes were blocked in PBS, 5% milk, 0.2% tween-20. Primary antibodies were diluted in this buffer and allowed to incubate overnight at 4°C. Secondary antibodies IR800 conjugated goat anti-rabbit (LI-COR Biosciences) were added at concentration 1/5000 at RT for 2 h. Visualization and quantification were performed using a LI-COR Odyssey and software (Odyssey 3.0.16) using median background subtraction. A minimum of three independent blots was run for each NET and control. The averages from all three are presented in figures with standard error shown.

### siRNA oligos

NET4/TMEM53 oligo si1: 5′-AGAAGUGGGUGUGGAAGAGGG-3′. NET4/TMEM53 oligo si2: 5′- UAGUAAGUAGGGUAGUCACGG-3′. Scramble control oligo 5′-UCGAAGUAUUCCGCGUACG-3′. Sigma NET4/TMEM53 esiRNA EHU027971.

pRb siRNA oligo: GCCCUUACAAGUUUCCUAG [49].

### RT-PCR analysis

Cells were lysed on tissue culture plates with Tri-Reagent (Sigma), and total RNA was extracted according to the manufacturers instructions. RT-PCR reactions were carried out with 100 ng of total RNA using the Titan one tube RT-PCR system (Roche) in accordance with the manufacturer's instructions, except that the dNTP concentration was increased to 500 mM and MgCl_2_ increased to 3 mM. Typical reaction conditions were 30 min reverse transcription at 50°C, 2 min denaturation at 94°C, then 24 cycles of 94°C for 30 s, 60°C for 30 s and 68°C for 45 s. Peptidylprolyl isomerase A (PPIA) was used as a loading control and reactions were repeated at least three times.

The human primer sets used were: NET4/TMEM53 5′-AAGCTGCTCGAGCTGCTC3-3′ and 5′-CAGAGGCTTGTGTAGTAA-3′; PPIA 5′-CACCGTGTTCTTCGACATTG-3′ and 5′-TCGAGTTGTCCACAGTCAGC-3′.
